# Exploring the Relationship between Access to Water, Sanitation and Hygiene and Soil-Transmitted Helminth Infection: A Demonstration of Two Recursive Partitioning Tools

**DOI:** 10.1371/journal.pntd.0002945

**Published:** 2014-06-12

**Authors:** Katherine Gass, David G. Addiss, Matthew C. Freeman

**Affiliations:** 1 Department of Epidemiology, Emory University, Atlanta, Georgia, United States of America; 2 Children Without Worms, Taskforce for Global Health, Decatur, Georgia, United States of America; 3 Department of Environmental Health, Emory University, Atlanta, Georgia, United States of America; University of Queensland, Australia

## Abstract

**Background:**

Soil-transmitted helminths (STH) – a class of parasites that affect billions of people – can be mitigated using mass drug administration, though reinfection following treatment occurs within a few months. Improvements to water, sanitation and hygiene (WASH) likely provide sustained benefit, but few rigorous studies have evaluated the specific WASH components most influential in reducing infection. There is a need for alternative analytic approaches to help identify, characterize and further refine the WASH components that are most important to STH reinfection. Traditional epidemiological approaches are not well-suited for assessing the complex and highly correlated relationships commonly seen in WASH.

**Methodology:**

We introduce two recursive partitioning approaches: classification and regression trees (C&RT) and conditional inference trees (CIT), which can be used to identify complex interactions between WASH indicators and identify sub-populations that may be susceptible to STH reinfection. We illustrate the advantages and disadvantages of these approaches utilizing school- and household-level WASH indicators gathered as part of a school-based randomized control trial in Kenya that measured STH reinfection of pupils 10 months following deworming treatment.

**Principal Findings:**

C&RT and CIT analyses resulted in strikingly different decision trees. C&RT may be the preferred approach if interest lies in using WASH indicators to classify individuals or communities as STH infected or uninfected, whereas CIT is most appropriate for identifying WASH indicators that may be causally associated with STH infection. Both tools are well-suited for identifying complex interactions among WASH indicators.

**Conclusions/Significance:**

C&RT and CIT are two analytic approaches that may offer valuable insight regarding the identification, selection and refinement of WASH indicators and their interactions with regards to STH control programs; however, they represent solutions to two distinct research questions and careful consideration should be made before deciding which approach is most appropriate.

## Author Summary

Soil-transmitted helminths (STH) are pervasive enteric parasites that lead to cognitive, nutritional and educational sequelae. Mass drug administration is employed to reduce morbidity, but reinfection occurs rapidly in the absence of changes to other environmental conditions, such as improvements to water, sanitation and hygiene (WASH). Since WASH behaviors and conditions are highly interrelated, typical epidemiological methods are limited. Few rigorous studies have assessed the impact of WASH components as they complement deworming and even fewer have sought to prioritize among the available indicators or identify complex interactions. In this paper we introduce two recursive partitioning approaches: classification and regression trees (C&RT) and conditional inference trees (CIT). We demonstrate these two tools using data from a school-based cluster-randomized trial conducted in Kenya. We discuss the advantages and disadvantages of each tool and give examples of how they may be used to improve STH control programs.

## Introduction

Infection with soil-transmitted helminths (STH), intestinal nematodes, is classified by the World Health Organization (WHO) as a neglected tropical disease (NTD). More than 1 billion people are infected and up to 5.3 billion are at risk of infection with at least one species of STH, including roundworm (*Ascaris lumbricoides*), whipworm (*Trichuris trichiura*), or hookworm (*Necator americanus* or *Ancylostoma duodenale*) [1]–[3]. STH infection occurs through fecal exposure, either through the skin in contaminated soil (in the case of hookworm) or ingestion of fecal material, typically in soil, on food, or on fingers [4]. Morbidity is most acute in school-age children, though high levels of hookworm infection can persist into adulthood [4]. It is estimated that between 5 and 39 million disability adjusted life years are lost due to STH infection [5], [6].

Though a recent review found limited evidence [7], STH infections have been found to impact on growth and nutrition of children [8] and reduce pupil absence in some studies [9], [10]. Control of STH is a priority for the WHO [11] and several countries, including Kenya, are scaling up mass drug administration in school-age children to reduce STH-related morbidity [12], [13]. These infections can be treated safely and effectively with the anthelminthic drugs albendazole or mebendazole [4], [14]. However, in the absence of improved access to water, sanitation, and hygiene (WASH), reinfection occurs and the prevalence and intensity of infection can reach pre-treatment levels in as few as six months, with 94% reinfection after 12 months [15]. Access to WASH includes hardware – such as toilet facilities that separate human feces, protected water supply, and soap – as well as behaviors, such as hand washing at key times and toilet use. The UNICEF and WHO Joint Monitoring Program (JMP) is the most widely cited source of data on what is considered “improved” water supply and sanitation [16], but the JMP does not provide guidance on hand washing, nor are its definitions specific to STH control. Even in countries with moderate access to improved water and sanitation in sub-Saharan Africa there is considerable geographic inequity [17]; these same marginalized populations without access are the ones with high risk of STH [18].

WASH components thought to be most critical for control of STH are the use of a clean toilet facility and the presence of water and soap for hand washing; however, few randomized trials have been conducted to assess the relationship between WASH and STH infection. Three randomized controlled trials have found evidence that improved hand washing with soap can lead to lower STH infection [19]–[21]. Nonetheless, in a study by Dumba et al., researchers did not find any impact of a participatory hygiene and sanitation transformation (PHAST) intervention compared with a control group that received deworming alone [22].

A recent meta-analysis of 36, mostly observational, studies suggested that access to and use of sanitation facilities is associated with significant reductions in the prevalence of STH infection, with an odds ratio [OR] of 0.54 (95% CI: 0.43–0.69) for *A. lumbricoides*, 0.58 (95% CI: 0.45–0.75) for *T. trichiura*, and 0.60 (95% CI: 0.48–0.75) for hookworm [23]. In a separate meta-analysis, soap use (OR: 0.53, 95% CI: 0.29–0.98), wearing shoes (OR: 0.38, 95% CI: 0.18–0.81) and drinking treated water (OR: 0.45, 95% CI: 0.36–0.58) were associated with lower STH infection [24]. Access to piped water was associated with lower infection with *A. lumbricoides* (OR:0.39, 95% CI: 0.39–0.41) and *T. trichiura* (OR: 0.57, 95% CI: 0.45–0.72). However, because nearly all studies in these meta-analyses were observational, it was not possible to disentangle the impacts of individual WASH components or the relationship between WASH and socio-economic status, potentially biasing many of these results.

WHO has set the goal of elimination of STH as a public health problem by 2020, which is provisionally defined as a prevalence of moderate- and high-intensity STH infection of <1% (WHO, 2012). To achieve this goal, and to sustain the gains made possible through mass drug administration, WASH improvements and intersectoral collaboration will be critical [11], [25]. However, identifying and characterizing those WASH components that are most effective at reducing or preventing STH infection is non-trivial, in part because of the ethical challenges of conducting randomized control trials which are necessary for establishing causal relationships [24], and yet will be essential for developing evidence on the success of STH control programs [18].

One challenge is that access to the different components of WASH in both the public and private sphere is highly interrelated, and little is known about the relative contributions of each independent WASH component in mitigating infection with STH. Furthermore, readily measurable WASH components relevant for STH control have not been identified or validated. Indeed, current WHO guidelines for STH control refer to WASH in general terms [11], [26]. The vast majority of studies examining the association between WASH components and STH infection have considered the main effects; however, because of the inherent connectedness of WASH components – e.g. water must be present for hand washing to occur – it is also critical to consider interactions. The number of potentially measurable WASH components is quite large, and when one also considers all the potential first, second-, and higher-order interaction terms, most datasets would not have sufficient power to detect all important associations using standard analytic approaches. A need exists to identify alternative analytic approaches to help identify, characterize, and further refine those WASH components that are most important to STH infection.

The goal of this analysis is to introduce two analytic approaches that are relatively new to the NTD and WASH communities: classification and regression trees (C&RT) and conditional inference trees (CIT). Both C&RT and CIT are a type of recursive partitioning, a nonparametric analytic approach well-suited for handling datasets with large numbers of predictor variables, identifying complex interactions, and selecting independent variables that are most predictive of or associated with the outcome [27]. These approaches are particularly useful for hypothesis generation and as a precursor to other model building approaches. We demonstrate how both methods can be applied to a dataset measuring household- and school-level WASH components and STH infection in Kenyan school children. This is a secondary analysis of the data; the primary results from this study have been reported elsewhere [9]. We discuss the relative merits and weaknesses of each approach and make recommendations for their uses.

## Methods

### Ethics Statement

Data collection for this study was approved by the Institutional Review Board at Emory University and the Ethics Committee at Great Lakes University of Kisumu (Kenya). We obtained a *loco parentis* from the head teacher at each school. Children provided oral consent to participate in this study, which was documented on the electronic data collection form. The ethics committees approved both a waiver of parental consent and the use of oral consent for study participants.

### Study Design

This study utilized data from a cluster-randomized trial to assess the impact of improved school and household WASH access on STH infection in Nyanza Province, Kenya from 2007–2009 [28]. Data for this analysis were collected in February 2009 – the final survey round of the trial – from 1,106 students in 39 public primary schools (Checklist S1: STROBE Checklist). Twenty of these schools had been randomly selected to receive a school-based WASH intervention that included construction of ventilated-improved pit latrine facilities at the school, hand washing and drinking water storage containers, teacher training on hygiene behavior change, and a one-year supply of dilute sodium-hypochlorite used for treatment of drinking water at the point of use. Pupils in all schools – both intervention and control – were dewormed at baseline (May, 2007) and midterm (April, 2008) using 400 mg of albendazole.

Pupils from grades 3 to 5 who were between the ages of 7 and 13 and had been dewormed during the previous round of data collection were randomly selected and enrolled into the original trial. The mean number of pupils was 302 and 275 in the intervention and control schools, respectively. This age group was selected because they experience the greatest burden of *A. lumbricoides* and *T. trichiura*, though peak morbidity for hookworm occurs later [29]–[31]. Systematic random selection of 30 pupils was conducted using a list of pupils from the school records, though some pupils were absent the day of the study. Only one child per household was enrolled to avoid the need to adjust for intra-household correlations. Of the 1106 students included in the study, 1095 provided a single analyzable stool and had valid Kato-Katz results. The original sample included pupils from 40 schools (20 intervention and 20 control). However, one control school was dropped from the analysis after children were treated with an additional round of deworming drugs.

Stool samples were collected and transported to the laboratory in cool boxes and examined microscopically within one hour of preparation using the Kato-Katz method [32]. Each stool sample was processed on two separate slides and read by different laboratory technicians to ascertain the eggs per gram of each STH species. Presence of infection was defined as detection of one or more eggs on either slide. Because all individuals were dewormed 10 months prior to the study, any infection observed was interpreted as incident infection. This analysis includes data on pupils from both intervention and control arms.

Data on individual demographics, household WASH conditions, and school WASH conditions were collected using structured observations and questionnaires. Pupils were interviewed to determine their age, sex, shoe wearing, comfort using the latrine at home and school, knowledge on hand washing and water supply treatment, opinion about latrine conditions, access to hand washing and drinking water at school, and their soil eating behavior (known as pica or geophagy), a common practice in western Kenya [33]. As a complement to the direct observations made at each school, pupil responses regarding school-based access to drinking water, hand washing water, soap, and latrines were aggregated at the school-level as an estimate of school WASH access.

One caregiver – typically the maternal head of household – for each pupil enrolled in the study was interviewed in his or her home to determine if one or both parents was alive and, if alive, the highest level of education achieved, socio-economic status through an asset index; access to an improved drinking water source, as defined by UNICEF and WHO [34]; if treated water is used; and the presence and condition of a household latrine. School head teachers were interviewed about the school's access to an improved water source during the dry season, pupil to latrine ratio, and latrine conditions. Enrollment data were taken from official school records.

In order to calculate socio-economic status, we used a principal component analysis (PCA) using assets observed at the household [35]. These assets included household construction materials, ownership of goods such as a TV and radio, and connection to electricity [36]. PCA was also used to construct an index of sanitation conditions at both the household and school, which included odor, presence of flies, presence of feces, wall material, condition of the slab, and presence of a functioning door. These components were put on a scale from 1–4 and the resulting value was a relativistic score of the average conditions for all latrines at the school or for the latrine at home. Two scores – latrine cleanliness and latrine structure – were derived based on factor loading. We did not include the score for latrine conditions at the home in our tree analysis, since that would limit the analysis to children with latrines at home. Acceptable latrines were classified as those for which no parameter scored in the lowest two values for each of the five sanitation categories.

### Statistical Analysis

This study compares two different recursive partitioning approaches: C&RT and CIT. Recursive partitioning is a nonparametric regression approach; it is a form of hierarchical clustering in which the data are sequentially split into dichotomous groups such that each resulting group contains increasingly similar responses for the outcome [37], [38]. Recursive partitioning has several advantages over traditional logistic regression. C&RT and CIT are supervised clustering approaches; they create partitions based on an outcome variable, as opposed to other clustering approaches such as k-means and PCA, which do not involve the outcome [39], . As nonparametric approaches, C&RT and CIT make no assumption of a monotonic or parametric relationship with the outcome, can be used to identify complex interactions among the independent variables without *a priori* specification of interaction terms, and can handle datasets where the number of independent variables is high relative to the number of observations. This final feature is particularly attractive to studies such as this, where a goal is to identify a few best predictors from many.

Both C&RT and CIT result in the formation of a decision tree with three levels consisting of a root node, internal nodes, and terminal nodes. Every tree starts with a “root node” that contains the sample of data from which the tree will be grown (e.g. the study population). The data are then partitioned into two “child nodes” based on the value the independent variable (IV) that best meets some partitioning criterion. The resulting child nodes each contain a subset of the original data. Each child node may be further partitioned, again based on the value of an IV. This process continues until no further partitions remain or some set of partitioning criteria are no longer met, resulting in terminal nodes. Terminal nodes, by definition, cannot have offspring. C&RT and CIT differ in the partitioning criteria used to select the IVs.

Under C&RT the data are partitioned according to the IV that results in the greatest improvement in the distribution homogeneity of the outcome [41], also referred to as reducing node impurity. Put another way, the data are split according to the IV that best improves predictive accuracy in the child nodes. The predictive accuracy of each potential binary split is considered independently and the split offering the greatest improvement is chosen to partition the data.

The initial tree generated by the recursive partitioning process of C&RT tends to be large (i.e. contain many splits of the data) and runs the risk of over-fitting the data. This motivates a second stage of tree construction called “pruning”, which can be viewed as analogous to backwards selection in linear regression. Through pruning, partitions of the data that are deemed to be the most superfluous are removed from the bottom-up. Cross-validation is then used to select the optimal sub-tree from the initial tree. In this study, C&RT analysis was performed using the ‘rpart’ package in R, version 2.13.2, available at http://cran.r-project.org/web/packages/rpart/index.html. For a more detailed description of this method see Therneau et al [42].

With CIT, the partitioning criterion is based on statistical significance and, unlike C&RT, accounts for conditional relationships between IVs. In the first step of the algorithm, the global null hypothesis of independence between all the IVs and the outcome is tested; if the null cannot be rejected, partitioning stops. If the global null hypothesis is rejected, then the IV that is the most significant in the model, conditional on the other covariates, is selected. When the selected IV is dichotomous, the choice of the best binary split is trivial; for non-dichotomous variables, the algorithm identifies the best binary split from all possible splits. Because CITs are based on statistical inference, pruning is not necessary. In this study, CIT analysis was performed using the ctree function in the ‘party’ package in R, version 2.13.2, available at http://cran.r-project.org/web/packages/party/index.html. See Hothorn et al for more information on this method [43].

All of the demographic and WASH indicators listed in Tables 1–3 were included in the analysis as IVs to be selected to partition the trees. The outcome of interest was any STH infection, coded dichotomously, with a “1” indicating the presence of at least one infection by *A. lumbricoides*, *T. trichiura*, or hookworm. Both C&RT and CIT trees were grown with the restriction that each node must have a minimum of 20 observations.

**Table 1:** Pupil-level independent variables included in C&RT and CIT analysis (n = 1095).

Independent Variable	Response	N (%)	Variable Type	WASH Characteristic
Age^1^	–	10.4 (1)^3^	Continuous	Demographic
Sex^1^	Female	575 (52%)	Dichotomous	Demographic
Geophagy at school or home^1^	Yes	138 (13%)	Dichotomous	Demographic
Shoes worn at school^2^	Yes	712 (65%)	Dichotomous	Demographic
School latrines dirty^1^	Yes	602 (55%)	Dichotomous	Sanitation
School latrines comfortable^1^	Yes	724 (66%)	Dichotomous	Sanitation
School latrines smell^1^	Yes	704 (64%)	Dichotomous	Sanitation
Child knows when to wash hands^1^	Yes	902 (82%)	Dichotomous	Hygiene

1 Reported by respondent (pupil).

2 Observed by interviewer.

3 Presented as the mean and standard deviation of the reported ages.

**Table 2:** Household-level independent variables included in C&RT and CIT analysis (n = 1095).

Independent Variable	Response	N (%)	Variable Type	WASH Characteristic
Mother's Education^1^	Deceased	133 (12%)	Ordinal	Demographic
	No education	423 (39%)		
	Primary	472 (43%)		
	Secondary or more	67 (6%)		
Father's Education^1^	Deceased	360 (33%)	Ordinal	Demographic
	No education	190 (17%)		
	Primary	324 (30%)		
	Secondary or more	221 (20%)		
District^2^	Kisumu East	211 (19%)	Categorical	Demographic
	Nyando	388 (35%)		
	Rachuonyo	507 (46%)		
Household asset score^3^	Asset score in lowest quintile	329 (30%)	Dichotomous	Demographic
Dry season improved water source^1^	Yes	537 (49%)	Dichotomous	Water
Hand washing station in home^2^	Yes	431 (39%)	Dichotomous	Hygiene
Household private latrine^2^	Yes	694 (63%)	Dichotomous	Sanitation

1 Reported by respondent (parent, typically mother).

2 Observed by interviewer.

3 Calculated using principal components analysis from the observed presence of household assets.

**Table 3:** School-level independent variables included in C&RT and CIT analysis (n = 39).

Independent Variable	Response	N (%)	Variable Type	WASH Characteristic
Intervention status^2^	Intervention group	20 (51%)	Dichotomous	WASH
	Control group	19 (49%)		
Pupil enrollment (2009)^3^	*Parameterized by quintiles*	NA	Ordinal	Demographic
Available drinking water^2^	Yes	23 (59%)	Dichotomous	Water
Available hand washing water^2^	Yes	23 (59%)	Dichotomous	Water
Current season improved water source^1^	Yes	27 (69%)	Dichotomous	Water
Dry season improved water source^1^	Yes	19 (49%)	Dichotomous	Water
		**Mean (SD)**		
Drinking water always available^4^	*Proportion of pupils responding* “*yes,*” *per school*	0.51 (0.4)	Continuous	Water
Hand washing water always available^4^	*Proportion of pupils responding* “*yes,*” *per school*	0.39 (0.4)	Continuous	Hygiene
Soap always available^4^	*Proportion of pupils responding* “*yes,*” *per school*	0.12 (0.1)	Continuous	Hygiene
Acceptable latrines per 100 pupils^5^	*Observation and school records*	1.03 (1.6)	Continuous	Sanitation
Latrine Structure^6^	*Observation and principal component analysis*	NA	Continuous	Sanitation
Latrine Cleanliness^6^	*Observation and principal component analysis*	NA	Continuous	Sanitation

1 Reported by respondent (head teacher); “improved” based on definitions by the WHO/UNICEF Joint Monitoring Program for Water Supply and Sanitation [34].

2 Observed by interviewer.

3 Obtained from school records.

4 Originally a yes/no variable reported individually by each pupil, then combined to get a percent of pupils reporting “yes” for each school.

5 Calculated using principal component analysis derived from observation and school enrollment records.

6 Calculated using principal component analysis derived from observation.

The C&RT results were validated using 10-fold cross-validation and the optimal tree was selected by pruning to the smallest tree within one standard error of the minimum cross-validated error tree [44]. Two different CITs were generated. In the primary analysis a minimum p-value of 0.05 was used for the partitioning criterion; in a secondary analysis, p-values were adjusted for multiple comparisons, using the Bonferroni correction.

## Results

### Study Population and WASH Characteristics

Table 1 contains the prevalence of key demographic and WASH variables measured at the pupil level. Over half (575; 52%) of the pupils surveyed were boys with a mean age of 10.4 years. Approximately a third (383; 35%) of pupils were observed without shoes at school and 138 (13%) reported some form of soil eating, known as geophagy. Pupils had a mixed impression of their school latrines, with 55% and 64% reporting the latrines to be dirty and have a strong odor, respectively; while 66% reported the latrines to be “comfortable.”

Table 2 contains information regarding the household demographic and WASH characteristics. The prevalence of orphanhood was high in the households surveyed, with 12% of mothers and 33% of fathers deceased. Of the households with living mothers, only 67 (6%) had completed at least secondary education and 423 (39%) had no formal education. Nearly half of the households had an improved water source in the dry season (537; 49%). The presence of a latrine was high (63%), though few hand washing stations were observed (39%).

Information regarding school-level WASH characteristics is in Table 3. Available water for drinking and hand washing was observed at the time of interview in nearly 60% of schools. Only half of the schools had an improved water source in the dry season, while approximately 70% had an improved source in the rainy season. The frequency with which pupils reported constant availability of water for drinking and hand washing at school varied widely between schools. Most students reported that soap was not always available at school.

Of the 1095 pupils tested by Kato Katz, 18% tested positive for at least one worm infection, with 81 pupils testing positive for *A. lumbricoides*, 75 for hookworm and 74 for *T. trichiura* (Table 4). Thirty-three children (3%) tested positive for two STH species. No samples were positive for all three worm types. More detailed information on the worm burden and main effects in this data have been previously published in Freeman et al [9].

**Table 4:** Helminth incidence^1^ by worm type (n = 1095).

Helminth Species	Positive Cases^2^ N (%)	Eggs per gram Geometric Mean^3^
*A. lumbricoides*	81 (7%)	2391
Hookworm *spp.*	75 (7%)	179
*T. trichiura*	74 (7%)	122
Any (of the three)	197 (18%)	–

1 Incidence because we are measuring reinfection following deworming.

2 By Kato-Katz.

3 Among positive cases.

### Classification and Regression Tree (C&RT)

Initial attempts to generate a classification tree failed to result in anything other than the root node (the starting dataset with no partitions) after pruning. This means that after cross-validation the tree generated showed no significant improvement in predictive accuracy over the starting dataset. A second C&RT analysis was performed, this time with sensitivity weighted more heavily than specificity under the assumption that in a situation of STH control, the identification of true STH infections is likely to be prioritized over true no infections. This was achieved by setting a misclassification cost of 2∶1 for STH positive vs. STH negative infections. Typically the C&RT algorithm tries to minimize the proportion of misclassified cases, where misclassification costs are taken to be equal for every case (e.g. those individuals with both positive and negative stool examinations for STH). With the 2∶1 weighting employed, misclassified positive individuals count twice as much as misclassified negative individuals.

**Figure 1:**
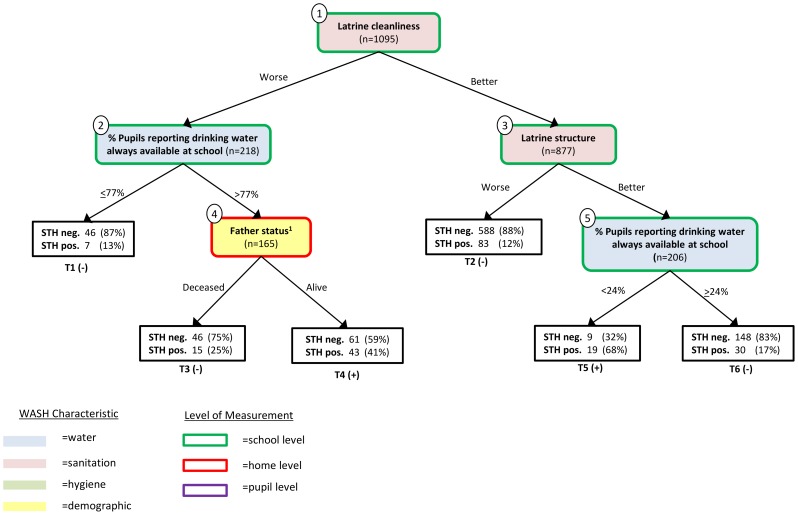
Classification tree for soil-transmitted helminth infection, with infection by any helminth (*Ascaris lumbricoides, Trichuris trichiura, *or hookworm *spp*.) considered to be STH positive. Each internal node contains the name of the independent variable (IV) selected to partition the data and the number of observations in the node. These nodes are numbered 1–5; the color of fill corresponds to the WASH characteristic of the IV and the border color represents how that characteristic was measured (e.g. at the school, home or pupil level). The branches emanating from each terminal node are labeled with the value of the IV used to partition the data. The square boxes represent terminal nodes and are numbered T1–T6 and contain the distribution of positive and negative cases at that terminal node as well as the predicted status for the node (“+” or “−“). Note that Terminal node T4 is classified as STH positive (“+”), even though the majority of pupils represented in the node are negative, because of the 2∶1 misclassification cost favoring sensitivity over specificity. 1This variable started out as a 4-level ordinal variable for father education but due to the optimal partition identified by the algorithm – “deceased’ vs. “no education”, “primary only”, and “secondary or more”– the variable ended up as indicator variables for father deceased.

The 2∶1 misclassification weighting resulted in a pruned classification tree with five partitions and six terminal nodes for predicting the incidence of infection by *any* of the three helminths. The following independent variables appeared in the final pruned tree: “latrine structure”, “latrine cleanliness”, “% pupils reporting drinking water always available at school”, and “father status” (Figure 1). The first split of the tree was the PCA factor for school latrine cleanliness, indicating that this variable was best at classifying STH infection status in the data. For both PCA-derived school sanitation variables appearing in the final tree (latrine cleanliness and latrine structure), the C&RT algorithm found an optimal partition of the PCA scores; however, because the numeric values of these scores have no external generalizability we relabeled the dichotomous groups as having “better” or “worse” sanitation conditions.

There were six terminal nodes in the final C&RT tree. Terminal node T5 had the greatest proportion of positive cases with 19 of the 28 children positive for one or more species of helminth; this terminal node corresponds to schools with good latrine cleanliness and structure but low reported drinking water availability. The C&RT algorithm labeled terminal nodes T4 and T5 as predictive of a “positive” STH infection, while the remaining four terminal nodes were predictive of no STH infection. Note that terminal node T4 is predictive of “positive” infection despite only 41% of observations being positive because of the 2∶1 weighting favoring sensitivity over specificity.

Table 5 shows the distribution of STH infection at each pair of child nodes emanating from the internal nodes in the C&RT analysis. Based on the classification tree, pupils with “worse” latrine cleanliness scores were twice as likely to be infected with STH (30%), compared to those with “better” latrine cleanliness scores (15%). Among pupils in schools with “better” latrine cleanliness scores, those with “better” latrine structure had twice the rate of infection (24%), compared to those with “worse” latrine structural conditions (12%). For schools with better latrine structure, greater values for “% pupils reporting drinking water always available at school” was predictive of lower levels of infection. Among those schools with “worse” latrine cleanliness and greater drinking water availability at school, the pupil-level variable for father's education status was identified as the best classifier of STH infection. The optimal partition of this pupil-level ordinal variable, which distinguished deceased fathers from living fathers with various levels of education (see Table 2), occurred between deceased fathers and living fathers regardless of education status.

**Table 5:** Distribution of STH infection at each branch of classification tree.

		Left Child Node *		Right Child Node *	
Internal Node #	Independent Variable	STH positive N (%)	STH negative N (%)	STH positive N (%)	STH negative N (%)
1	Latrine cleanliness	65 (30%)	153 (70%)	132 (15%)	745 (85%)
2	% pupils reporting drinking water always available at school	7 (13%)	46 (87%)	58 (35%)	107 (65%)
3	Latrine structure	83 (12%)	588 (88%)	49 (24%)	157 (76%)
4	Father status	15 (25%)	46 (75%)	43 (41%)	61 (59%)
5	% pupils reporting drinking water always available at school	19 (68%)	9 (32%)	30 (17%)	148 (83%)

*See Figure 1.

### Conditional Inference Trees (CIT)

**Figure 2:**
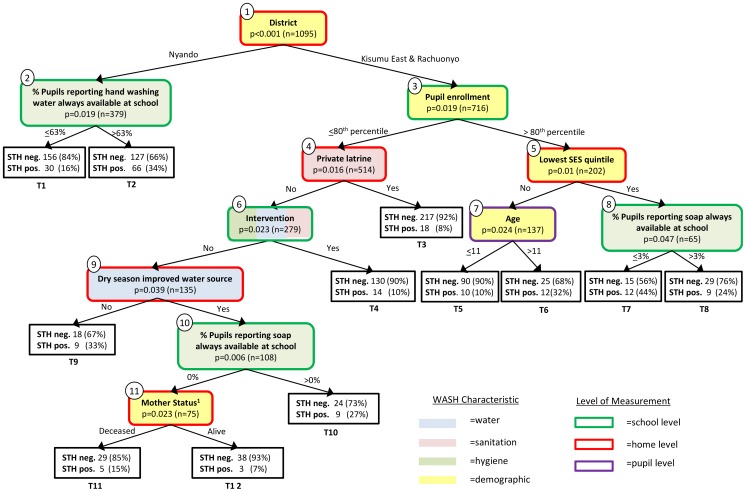
Conditional inference tree for soil-transmitted helminth infection, with infection by any helminth (*Ascaris lumbricoides, Trichuris trichiura,* or hookworm *spp*.) considered to be STH positive. Each internal node contains the name of the independent variable (IV) selected to partition the data, the p-value for the significance of the IV in the model, and the number of observations in the node. These nodes are numbered 1–11; the color of fill corresponds to the WASH characteristic of the IV and the border color represents how that characteristic was measured (e.g. at the school, home or pupil level). The branches emanating from each terminal node are labeled with the value of the IV used to partition the data. The square boxes represent terminal nodes and are numbered T1–T12 and contain the distribution of positive and negative cases at that terminal node. 1This variable started out as a 4-level ordinal variable for mother education but due to the optimal partition identified by the algorithm – “deceased’ vs. “no education”, “primary only”, and “secondary or more”– the variable ended up as indicator variables for mother deceased.

The primary CIT tree, generated without adjustment for multiple comparisons, had 12 terminal nodes (Figure 2). The IV most significantly associated with STH infection, conditional on all other variables in the model, was “District”. Of the 11 IVs appearing in the tree, 5 were measured at the schoollevel, 5 at the household level and 1 (“age”) at the pupil level. Five of the IVs in the final tree were demographic measures while the remaining six were WASH indicators. Terminal node T7 had the greatest proportion of positive cases with 44% (n = 12) of individuals testing STH positive. This node corresponds to pupils from Kisumu East or Rachuonyo Districts with low family SES who attend a school with high enrollment rates and little to no soap reportedly available.

Table 6 shows the distribution of STH infection for each pair of child nodes emanating from the internal nodes in the CIT analysis. In Nyando District, greater reported availability of water for hand washing in the schools was associated with a *greater* incidence of STH infection (34% vs. 16%, from terminal nodes T1 and T2). In the Kisumu East and Rachuonyo Districts, among those schools with high enrollment rates (>80^th^ percentile), pupils whose household was in the lowest SES quintile had twice the incidence of STH infection (32% vs. 16%; Table 6). The IV “% pupils reporting soap always available at school” appeared twice in the tree in Figure 2 (internal nodes 8 & 10) but with opposite directions of association; at internal node 8, little to no soap availability was associated with increased STH infection (44% vs. 24%), whereas at internal node 10 lack of soap was associated with decreased STH infection (11% vs. 27%).

**Table 6:** Distribution of STH infection at each branch of conditional inference tree.

		Left Child Node *		Right Child Node *	
Internal Node #	Independent Variable	STH positive N (%)	STH negative N (%)	STH positive N (%)	STH negative N (%)
1	District	96 (25%)	283 (75%)	101 (14%)	615 (86%)
2	% pupils reporting hand washing water always available at school	30 (16%)	156 (84%)	66 (34%)	127 (66%)
3	Pupil enrollment	58 (11%)	456 (89%)	43 (21%)	159 (79%)
4	Private latrine	40 (14%)	239 (86%)	18 (8%)	217 (92%)
5	Low SES	22 (16%)	115 (84%)	21 (32%)	44 (68%)
6	Intervention	26 (19%)	109 (81%)	14 (10%)	130 (90%)
7	Age	10 (10%)	90 (90%)	12 (32%)	25 (68%)
8	% pupils reporting soap always available at school	12 (44%)	15 (56%)	9 (24%)	29 (76%)
9	Dry season improved water source	9 (33%)	18 (67%)	17 (16%)	91 (84%)
10	% pupils reporting soap always available at school	8 (11%)	67 (89%)	9 (27%)	24 (73%)
11	Mother status	5 (15%)	29 (85%)	3 (7%)	38 (93%)

*See Figure 2.

**Figure 3:**
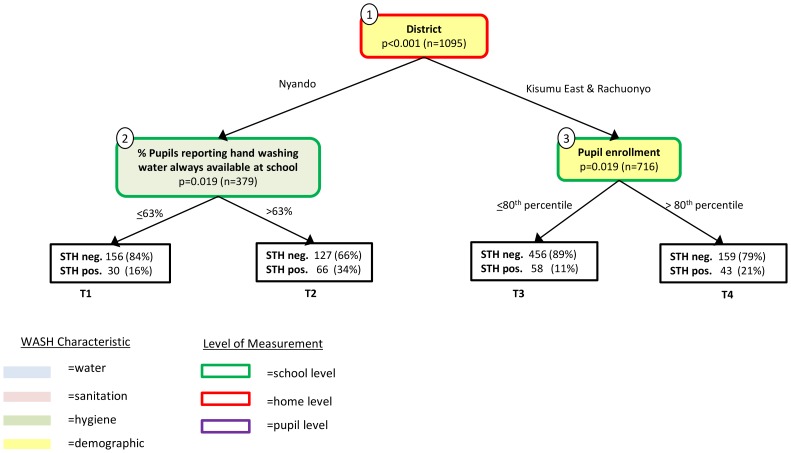
Conditional inference tree for soil-transmitted helminth infection with p-values adjusted for multiple comparisons. This is a sub-tree of the tree in Figure 2. The p-values shown in each internal node are adjusted for multiple comparisons.

The second conditional inference tree, grown with p-values adjusted for multiple comparisons, is shown in Figure 3. This tree is a sub-tree of the tree in Figure 2 and represents a more conservative approach. The tree in Figure 3 has four terminal nodes, with the greatest STH incidence seen in the terminal node for pupils in Nyando District attending schools where more than 63% of the students reported hand washing water available (34% of pupils in this node were positive for STH).

The branching of the IVs in both the classification and conditional inference trees can be used to identify potential interactions between WASH indicators that may be important predictors of STH infection. The classification tree illustrates that when latrine cleanliness is better, latrine structure is an important determinant of STH infection. In this instance, worse latrine structure predicts lower STH infection (Figure 1). When both latrine cleanliness and structure are good, the pupil-reported presence of water at school is important, with poor water availability (<24% of students reporting constant water availability) associated with higher STH infection (T5, Figure 1) and more consistent water availability predicting less STH infection (T6, Figure 1). The conditional inference tree suggests that the interaction of living in the Nyando District and the reported availability of hand washing water at school are together associated with STH infection (Figure 2). Similarly, for those living in the Kisumu East and Rachuonyo Districts the CIT analysis identified an interaction between pupil enrollment, low SES and age (among other interactions present in the tree).

## Discussion

In the past several years classification and regression trees, first introduced by Breiman et. al. in 1984 [45], have started to gain recognition as a statistical tool in NTD research. C&RT is most commonly used in the NTD research community as a tool for disease prediction and classification [46]–[48], as well as to identify the hierarchical importance of predictor variables [49], [50]. To our knowledge there have been no studies that have used C&RT or CIT to examine WASH data in relationship to enteric disease prevalence or incidence, specifically STH. Given the policy and programmatic interest in quantifying the impact of WASH on STH, these recursive partitioning approaches may prove to be important analytic tools.

In this study we show how C&RT and CIT can be used as tools to better understand household- and school-level WASH indicators and their relationship with STH infection in children. While both C&RT and CIT result in the generation of a decision tree, the dissimilarities between Figures 1 and 2 make it clear that these two approaches are not identical. Instead C&RT and CIT represent solutions to two distinct research questions and careful consideration should be made before deciding which tool is most appropriate.

A C&RT analysis is well-suited when the goal is prediction or classification. Independent variables are chosen to partition the data according to the one that results in the biggest improvement to predictive accuracy, and not according to association with the outcome, as in the CIT analysis. The difference in predictive performance between these two types of trees is apparent when comparing the sensitivity and specificity of the classification tree (Figure 1) with the conditional inference tree (Figure 2), where observations in each terminal node of the CIT are classified according to the majority of observations in that node (e.g. if ≥50% of pupils in the terminal node are STH positive, then all individuals in that node will be classified as “positive”). In Figure 1, of the 197 children testing positive for STH infection, 62 were correctly classified by the tree, for a sensitivity of 31%; of 898 children who tested negative, 828 were correctly classified, resulting in a specificity of 92%. By contrast, in Figure 2, *all* of the terminal nodes had fewer than 50% positive cases, resulting in a sensitivity of 0% and a specificity of 100%. It is important to keep in mind that the C&RT results were generated with a preference towards sensitivity (using a 2∶1 misclassification cost), which is why terminal node T4 is classified as “positive” despite only 41% of cases being positive. This weighting was necessary to obtain a final pruned tree that was more than just the root node. If we ignore this weighting and classify node T4 as “negative” according to the majority of observations, then the sensitivity of the C&RT tree falls to 10% and the specificity grows to 99%.

While one might expect CIT results to have poorer predictive accuracy, the poor predictive accuracy of the C&RT results is surprising and may have more to do with our data than the method itself. Firstly, the dataset used had relatively few positive cases (18% positive overall), making classification more difficult. Secondly, although infection was measured at the pupil level, nearly half of the IVs (12 of 27) were school level. While the presence of school-level IVs in the final tree could mean that school-level factors may play a greater role in driving STH infection than household factors, it also leads to some major drawbacks. In Figure 1, terminal nodes T1, T2, T5 and T6 are not classifying STH infection beyond the school level; a predicted STH status of “positive” or “negative” is assigned to all students in the same school because there are no individual-level WASH factors to further differentiate pupils within the same school. Only among the subgroup of pupils in Figure 1 for whom “father status” is predictive of STH infection are we able to classify infection at the pupil level. As a result, the sensitivity of the STH tree is limited by the sensitivity obtained at the school level. Taken together, the scarcity of positive cases and use of school-level variables likely explain the failure of the initial C&RT analysis to identify anything beyond the root node. A further limitation of using data clustered at the school level is that to our knowledge there is no way to account for the design effect (decrease in the sample variance due to clustered sampling) in either the C&RT or CIT analysis packages. This may affect the validity of the CIT results if the design effect varies by IV.

C&RT may be the preferred approach if interest lies in using WASH indicators to classify individuals, or communities, as STH infected or uninfected. Under this framework, a representative dataset would be used to grow a classification tree (as was done in this analysis), which could then be used to predict the STH status of future data collected from individuals or communities. One potential application of this approach is the development of a rapid screening tool for classifying communities as “likely endemic”, “likely sub-endemic” or “likely non-endemic” for STH based on the values of the set of IVs in the classification tree. Such an approach might lessen the initial need for specimen collection and conserve resources.

When the goal is to identify the IVs most associated with the outcome, in order to estimate causal effects, conditional inference trees are the more useful tool. At each branch of a conditional inference tree the IV that is most significantly associated with the outcome is chosen to partition the data, resulting in a tree built on statistical significance. An advantage of CIT over traditional regression is that, as a nonparametric approach, it can identify non-linear associations in the data. A CIT analysis can be used as a form of variable selection, identifying the few IVs that result in the most significant main and joint effects. Such an approach would be useful when trying to determine which of the many WASH indicators to include in an intervention package in order to see the greatest impact on STH incidence.

Both C&RT and CIT are useful as exploratory analytic tools for identifying complex interactions in the data. Using standard analytic approaches to assess interaction often requires including the product of two or more predictors in the same model and assessing whether the resulting coefficient differs significantly from zero. As the number of potential predictors increases, the number of potential second-, third- and higher-order interactions grows, becoming too large to include in any one model without substantial *a priori* knowledge about complex interactions [51]. A C&RT or CIT analysis allows one to move beyond the commonly reported main effects, to identify complex interactions (e.g. non-linear, multi-order interactions) in the data. Often the interactions identified by the branching of the tree are ones that might not have been identified *a priori* by the researcher (e.g. the interaction of district, pupil enrollment, low SES and age found in Figure 2) and can be used to generate hypotheses leading to further research. These interactions can also be used to identify potentially vulnerable sub-populations. For example, the tree in Figure 2 suggests that children in Nyando District who attend schools with greater reported prevalence of hand washing water have a greater risk of STH infection – a surprising and somewhat paradoxical finding. It is important to interpret any interactions in the context in which they were generated, namely for prediction (C&RT) or association (CIT).

As with any analysis, it is important to consider the data and potential for biases when interpreting results. One challenge is that many WASH interventions are highly correlated. Research in other fields has shown that when two IVs are highly correlated, the effect estimate of the better measured variable will capture some of the effect of the less-well measured variable, making the overall effect estimates less accurate [52], [53]. This is of potential concern to WASH analyses where the potential for measurement error is high, due to the sensitivity of the topics and the way in which some indicators are measured (e.g. reported vs. observed measures). Examples of highly correlated variables in our analysis are “% pupils reporting soap always available at school”, “% pupils reporting hand washing water always available at school” and “intervention”. Because the intervention involved making soap and water available in some schools, these three variables are highly correlated (rho = 0.71, p<0.0001) in our data and the presence of any one of these in the final tree is likely capturing, in part, the effect of the others. One advantage of a CIT analysis is that IVs are selected conditional on all other variables in the model. That is, each partition represents the variable most strongly associated with STH infection *controlling* for the other IVs (i.e., WASH indicators) in the model. By simultaneously controlling for the other IVs, a CIT analysis alleviates many of the concerns with correlated data that are particularly pertinent to WASH analyses, though it does not resolve concerns about measurement error.

Another advantage of CIT over C&RT is that the former can handle independent variables with different types of classification (e.g. continuous, categorical, ordinal, etc.) without bias [43]. Studies have shown that C&RT favors continuous IVs over dichotomous or ordinal IVs [54], [55]. It may be that the appearance of “latrine cleanliness” and “latrine structure” in the first splits of Figure 1 has more to do with the fact they are among the few continuous IVs eligible to be selected for splitting and were unduly favored by C&RT. That neither of these two best classifiers in the C&RT analysis appeared in the CIT analysis is somewhat surprising (if they are truly good classifiers one would also expect them to be strongly associated with STH infection) and may instead be indicative of this bias towards favoring IVs with many possible splits. The optimal splits for “latrine cleanliness” and “latrine structure” could have been selected because the C&RT algorithm was able to identify a partition that most closely approximated some unmeasured risk factor for STH.

This is not to say that continuous variables should not be used in C&RT. Another way that C&RT is being used by the NTD community is to identify the best dichotomous cutoffs for continuous IVs. Martinez et. al used C&RT to find optimal cutoff for chromosome counts used in leprosy diagnosis while Levecke et. al utilized C&RT to find cutoffs for study design factors for the fecal egg count reduction test to monitor STH drug efficacy [56], [57]. It should be noted that CIT can also be used to identify optimal cutoffs associated with the outcome. A main effects analysis, using logistic regression, found neither of the principal components for latrine structure and cleanliness to be strongly associated with overall STH infection—which explains why they did not appear in the CIT analysis—but when dichotomized according to the cutoffs identified by C&RT (from Figures 1) the resulting indicator variables were highly significant in the regression analysis (results not shown).

While the goal of this study was primarily methodological, it is important to discuss some of the findings, particularly those that were surprising and at times counter-intuitive. In the first split of the C&RT tree, children in schools with worse latrine cleanliness had twice the incidence of infection compared to those with better latrine cleanliness (30% vs. 15%), which is in-line with our expectations. One example of a counterintuitive finding in the C&RT analysis is that among schools with better latrine cleanliness, better latrine structure is associated with *greater* infection. It is possible that latrines with better structure are more likely to be used, and even good cleanliness was insufficient to prevent infection. Another counter-intuitive finding from the C&RT results was that among schools with poor latrine cleanliness, poorer water availability is associated with *less* STH infection (Table 5, node 2). One reason for this surprising finding could be that the causal direction of association is not well-understood. Is the latrine cleanliness poor because the latrines are often used [58], or does poor cleanliness prevent the latrines from being used? Another surprising finding is that having ones' mother alive is predictive of *less* STH infection in the CIT analysis (Figure 2, terminal nodes T11 vs T12) while having ones' father alive is predictive of *more* STH infection in the C&RT analysis (Figure 1, terminal nodes T3 vs T4). While it is possible that there is a causal explanation behind this, it is also important to remember that the subsets of data used to make these split selections are likely to be quite different, based on the previous partitions leading to the nodes. The variable “% pupils reporting soap always available at school” appears twice in Figure 2 but suggests opposite directions of association. This could be due in part to the complex interactions leading up to these two terminal nodes. For example, by looking at the previous branching of the tree in Figure 2, one can see that node 10 contains schools that did not receive the intervention whereas the population in node 8 contains both intervention and non-intervention schools. It is possible that students in node 8 attending an intervention school that *should* have received soap, but report that it did not, are somehow more susceptible to STH infection. These, and other unexpected findings, further support our suggestion that these two approaches be used for hypothesis generating and that follow-up analyses be conducted to better understand these interactions identified in the data and determine if they are generalizable to other populations.

Another way of analyzing the trees in Figures 1 and 2 is by assessing the WASH characteristics of the IVs included at each split. In the classification tree, two of the IVs selected to partition the tree are related to water, two to sanitation and one is demographic; no indicators for hygiene appear in the C&RT tree. By contrast, 3 of the 11 independent variables in the conditional inference tree are indicators of hygiene, 1 of sanitation and 1 of water (and 1, “intervention” encompassing all three WASH components); the remaining 5 IVs selected are demographic indicators. These differences between the types of WASH indicators present in the two trees again serve to highlight the differences in the two approaches of growing trees: prediction vs. statistical significance. The trees are also suggestive of the complex relationship between demographic, water, sanitation and hygiene variables and STH infection; both trees involve many of these different variables with no single variable or class of variable emerging as the most related to STH infection.

The differences in the Figure 1 and 2 results also emphasize one of the common criticisms of recursive partitioning algorithms, that any single tree is often highly unstable [37]. With only 1095 observations in the entire dataset, tree stability is a definite concern in this analysis. Increasing sample size or incorporating multiple datasets to grow and test the trees can help to improve tree stability. Random forests is an alternative approach in which an ensemble of classification or conditional inference trees are grown and the results combined to get more stable measures of variable importance [38]; however a downside is that random forests do not result in a single tree diagram and cannot be readily used to identify complex interactions.

In addition to tree stability, the small sample size will also limit the power and generalizability of any one tree. With small datasets one runs the risk of generating trees that over fit the data, and thus any substantive findings may have limited applicability beyond the data. Cross-validation and splits based on statistical inference help to limit this concern in C&RT and CIT, respectively. Nonetheless, we caution the reader against over-interpreting the substantive findings in this paper. Moving forward, as issues related to WASH take on greater priority, we expect larger datasets to become available that will increase both the relevance and utility of these analytic approaches. A good example is the Global Trachoma Mapping Project, which, in addition to measuring trachoma prevalence, has collected data on water and sanitation indices on over half a million people [59].

A limitation of the data used in this analysis is that two of the independent variables that appeared in the final classification tree are data-specific; the indicators for latrine cleanliness and structure were created from two principal components. Because these variables are derived from the data they may not be generalizable to other studies and their numerical value has little direct interpretation or value as a metric; however, our findings regarding the direction of effect and relative importance of these characteristics remain valid. This analysis also highlights the need to arrive at methods for measuring these and other water and sanitation indicators that are feasible, replicable and generalizable.

### Conclusions

C&RT and CIT are two analytic tools that may be of use to the NTD and WASH communities, depending on the research objective. When prediction of the outcome is the goal, C&RT is likely to be the most favorable tool, whereas CIT is good for identifying the IVs most significantly associated with the outcome. Both methods can be used to identify complex interactions in the data; however, these interactions should be interpreted in the context of the tool (e.g. prediction vs. association). These interactions can then be incorporated into subsequent parametric analyses or used to generate hypotheses for future research. This study supports the WHO's goal for STH elimination by contributing to the research on the impacts of WASH in mitigating STH infection. With the help of this and future research it will hopefully be possible to identify the WASH indicators of greatest relevance for STH control.

## Supporting Information

Checklist S1 STROBE checklist.
(DOC)
